# Tackling the readmission epidemic: a resident teaching service perspective

**DOI:** 10.3402/jchimp.v2i4.19647

**Published:** 2013-01-07

**Authors:** Ravi K. Sharma, Pradeep Dhakarwal, Janelle Violago, Robin Bhasin, Ibrahim Ghobrial

**Affiliations:** University of Pittsburgh Medical Center, Department of Medicine, UPMC–Mckeesport

**Keywords:** hospital readmission, hospital quality, discharge planning

## Abstract

**Background:**

Readmission rates are projected to serve as quality measures that have the potential to negatively impact hospital and physician reimbursement. Individual physicians and hospitals are developing plans to reduce readmission rates. Successful plans should be based on specific data obtained from each individual type of practice.

**Objective:**

To analyze the etiological factors responsible for readmissions to various teaching services in a community hospital. This will serve to identify potentially correctable factors that will be the basis for developing practice-specific plans to reduce readmissions.

**Design:**

Retrospective detailed chart review.

**Setting:**

Community teaching hospital affiliated with a large academic health care system.

**Participants:**

Patients admitted to teaching services at a community hospital.

**Measurements:**

Data are presented as descriptive analysis.

**Results:**

Advanced chronic medical conditions (31%), patients’ lifestyle choices (28%), and new unrelated diagnoses (21%) are the major causes of readmissions. The remaining small percentage of readmissions is attributed to premature discharge, poor discharge planning, poor post-discharge follow-up, medication errors, and failure to implement medical care guidelines.

**Limitations:**

Retrospective study from a single center.

**Conclusions:**

Causes of readmission are diverse. Although most are universal, the relative contribution of each factor is unique to each population. Institutions should generate their own data in order to direct their resources toward ‘high return’ areas. Current studies emphasize the role of physicians and health systems in reducing readmission rates. However, the area of readmissions related to patients’ behaviors is not well explored. Our study identified the role of patients’ lifestyle choices as a major cause of readmission.

A readmission is defined by the Centers for Medicare & Medicaid Services (CMS) as a return hospitalization that follows an acute care admission within 30 days. Readmissions are common and add to the cost of health care. Soon, hospitals and physicians may receive reduced payments for care rendered for readmission in cases of congestive heart failure (CHF), myocardial infarction, and pneumonia (1). In 2007, 18% of Medicare patients discharged from hospitals were readmitted within 30 days, accounting for $15 billion in spending (2). Previous studies do not highlight the unique pattern related to teaching services. Interventions have been suggested and implemented but detailed analysis of the causes of readmission is lacking. Hence, despite several efforts, the impact on readmission rates remains poor (3–6) with exceptions of few positive results at single centers (7–11).

We initiated this multiphase project to: (a) identify the exact causes for readmissions in our patient population, (b) develop a risk assessment tool to identify high-risk patients at the time of admission, thereby directing our resources to the areas of highest yield, and (c) develop specific and practical interventions based on our population needs.

The objective of this first phase of the study is to analyze the etiological factors responsible for readmission in the patient population served by teaching services at our hospital.

## Material and Methods

### Data source, Abstraction, and Quality Assessment

Patients were eligible for the study if they were readmitted within 30 days of discharge to a teaching team at our institution between September 2010 and May 2011. Data were obtained from the quality office that monitors readmissions data. The study was approved by the quality improvement arm of the institutional review board (IRB) of the health system.

For each readmission episode, health records were reviewed. This included admission history and physical documents, daily progress notes, discharge summaries, emergency department notes, nursing and physical therapist documents of index admission and readmission, as well as post-discharge office notes. Data extraction was event based (i.e., multiple readmissions for one patient were evaluated separately). In total, 201 readmission events were analyzed.

Causes of readmission were categorized into nine classes as listed in the following section. The authors designed and finalized the protocol for chart review. Consistency of methodology was confirmed. Sample cases were discussed as a group, and feedback was provided. The same method of chart review was adopted by all authors to ensure consistency in data collection. Random cross reviews were done by the supervising reviewers, and no discrepancy was identified.

### Data Synthesis and Analysis

Readmission causes were categorized into nine different classes: 1) patients’ behavior-related factors, which include non-compliance with medications, treatments (including dialysis), dietary recommendation, or lifestyle choices (e.g., continuing recreational drug or alcohol use), 2) end-stage diseases (e.g., severe COPD, refractory heart failure, metastatic cancer, etc.), 3) medication-associated factors, which include financial reasons (cost and insurance coverage) as well as prescription errors, 4) premature discharges, which include patients who were unstable at discharge, 5) inadequate discharge instructions about diet, medications, follow-up, and so on, 6) failure of the discharging physician to provide the patient with a post-discharge office follow-up appointment within one week, 7) discharge to inappropriate level of care, 8) inappropriate return from nursing or personal care homes due to failure of timely evaluation by receiving physician, and 9) new medical diagnosis unrelated to the index admission.

Readmission data were organized in descriptive statistics. Patient demographics are tabulated in Table 1.

**Table 1:** Pertinent patient demographics

Patient demographics	
Gender	
Female	110 (54.7%)
Male	91 (45.3%)
Mean age	
Female	71.51±15.7
Male	61.51±14.8
Race	
White	152 (75.6%)
African American	49 (24.4%)
BMI (kg/m^2^)	
<25	73 (36.3%)
≥25≤35	85 (42.3%)
>35	43 (18.7%)
Disposition	
Home	116 (57.7%)
SNF/PCH/LTAC	85 (42.3%)

SNF, skilled nursing facility; PCH, personal care home; LTAC, long-term, acute care facility.

## Results

For a period of nine months from September 2010 to May 2011, 201 readmission events were analyzed. Causes of readmissions were diverse (Table 2). Severe end-stage illnesses, patient's behavior-related choices, and unrelated new diagnoses were the leading factors contributing to 31%, 28%, and 21% of readmissions, respectively. System-related modifiable causes include: medication-related problems (e.g., cost, formulary coverage, and prescription errors) (4%), premature discharge (3%), and early return from nursing home (2.5%). A small percentage of readmissions were attributed to inadequate discharge instructions (2.5%), physician-related poor follow-up post-discharge (6%), and discharge to inappropriate level of care (2%).

**Table 2:** Common conditions that led to readmissions

Factors	Frequency	Percent distribution
Chronic medical condition	90	31
Patient's behavior-related factor	79	28
New diagnoses (unrelated to the index admission)	59	21
Poor follow-up: home care, office visit	17	6
Medication problem (no coverage, reconciliation)	12	4
Unstable at discharge, inadequate treatment	9	3
Inadequate discharge instructions	7	2.5
Inappropriate discharge level of care	6	2
Nursing home premature return	7	2.5
Total frequency	286*	
Total chart reviewed	201	

* Total frequency of 286 in 201 readmission events suggest multiple factors contributed to each readmission event.

Major comorbid conditions influence hospital courses and complicate post-discharge outcomes. These include cardiovascular disease (congestive cardiac failure, coronary artery disease, peripheral arterial disease, arrhythmia, and venous thromboembolism), cerebrovascular events, chronic obstructive pulmonary disease, chronic renal insufficiency, including end-stage renal disease (ESRD), cirrhosis, connective tissue diseases, dementia, diabetes mellitus, hypertension, chronic ulcers, and malignancy. We observed that 37.3% (n = 75) of readmission episodes occurred when patients had 0–2 comorbidities while 62.7% (n = 126) readmissions occurred when patients had three or more comorbid conditions. In accordance with prior studies, comorbid conditions were associated with high readmission rate even if compensated at the time of the index admission. Detailed specification of comorbid conditions and their distribution in the study population is demonstrated in Table 3.

**Table 3:** Prevalence of comorbid conditions

Comorbid condition		Percent distribution
Cardiovascular		
Coronary artery disease (CAD)	50	24.87
Congestive heart failure (CHF)	60	29.85
Arrhythmias	53	26.36
DVT/PE	21	10.44
Peripheral arterial disease (PAD)	14	6.96
Chronic obstructive pulmonary disease	67	33.33
Cerebrovascular events	38	18.90
Connective tissue	8	3.98
Chronic renal insufficiency (including ESRD)	38	18.90
Cirrhosis	16	7.96
Diabetes mellitus	65	32.33
Dementia	12	5.97
Malignancy (active and treated)	38	18.90
Chronic ulcer	9	4.47
Hypertension	122	60.69
Drug/alcohol abuse	22	10.94

DVT, deep vein thrombosis; PE, pulmonary embolism, ESRD, end-stage renal disease.

## Discussion

Although our findings confirm the known risk factors for readmission, they shed more light on the proportional contribution by each factor and the potential for prevention and modification.

The finding that 28% of our readmissions are due to patient behavioral factors is very significant. Patient behavioral factors are related to lifestyle choices despite physicians’ advice. Examples include continuing drug misuse (e.g., repeated admissions for alcohol-related conditions, cocaine-related chest pains, frequent asthma, and COPD exacerbations related to smoking), missing dialysis, and subsequent fluid and electrolyte problems. Policymakers need to be aware of the obvious finding that readmissions do not always reflect poor quality of care (12); hence, placing the entire burden on health care providers is inappropriate. Policymakers and insurers should introduce patient incentives to reduce readmission. Suggestions include reducing insurance premiums or waiving co-payments for those who follow recommendations. Excluding patent-related behavioral factors, 20% of the readmissions are probably preventable.

In our study, 21% of the events were due to unrelated diagnoses. With this in mind, punitive measures against hospitals and physicians are unwarranted. Extensive review of the causes and circumstances of each readmission should be carried out before imposing such punitive measures. Due to the retrospective nature of the study, it is not possible to identify whether such ‘new readmission diagnoses’ were present and missed at the index admission. This is very unlikely because we studied patients in the teaching services where patients’ information is available in an extensive electronic health record system and patients receive comprehensive exams by multiple providers.

According to earlier studies, readmission rates are affected by the disease severity and comorbid conditions (13). Our study has similar results in that about 63% readmission events had three or more comorbid conditions. Comorbid conditions not only complicate hospital course but also play a significant role in recuperation from an illness. Adequate attention should be directed to comprehensive management of comorbid conditions.

Teaching teams are more likely to deal with difficult patients than private physicians because the latter are more likely to dismiss such patients who drain their resources and negatively impact their performance metrics. These patients usually end up on the teaching teams and should payments be reduced or withheld for such readmissions, teaching services will be disproportionately impacted. Senior managers of hospitals, health care systems, and integrated delivery and finance systems (IDFS) also need to be aware of these results to develop integrated systems for dealing with such patients (e.g., develop drug rehabilitation programs in proximity to hospitals with high prevalence of drug- and alcohol-related illnesses). Currently, there is a significant shortage of drug and alcohol rehabilitation facilities, and patients must often wait months for an appointment. We commonly observe patients relapsing into recurrent drug use before being seen at rehabilitation facilities. Resources should be directed into training and hiring more physicians with an interest in addiction medicine. It is very notable that only a small fraction of the tobacco settlement payments were invested in smoking cessation programs.

Rosenow reported that up to 45% of elderly patients may be non-compliant with medications because of inability to read or understand the labels (14). We observed that a significant portion of medication-related readmissions is preventable. Attention to cost and affordability, review of formulary status of discharge prescriptions, appropriate communication with pharmacies, arranging home delivery of medications, providing free short supply of medications for uninsured patients, providing adequate discharge instruction to patients and caregivers, planning home care visiting nursing, and early phone or office follow-up will likely help enhance compliance. Although time consuming, such detailed interventions are feasible with the help of a discharge nurse and a hospital pharmacist.

Poor discharge planning played a small role in readmissions in our study population: only 2.5% of readmissions were due to inadequate discharge instructions, 6% were due to sub-optimal follow-up, and another 4% were due to problems with medication coverage and reconciliation. Comprehensive discharge planning and longitudinal care provided by the teaching teams have kept these rates relatively low. These findings support the notion that improved discharge planning and follow-up care reduce the readmission rates at dedicated centers (15, 16). Other factors such as premature discharge (3%), premature return from nursing homes (2.5%), and inappropriate level of discharge (2%) played a small role, making a total of 20% readmissions due to preventable factors. This is consistent with the study by Van Walraven et al. that attributed 27% readmissions to preventable causes (17).

Patients with advanced medical conditions receiving end of life care constitute another major section of the readmission pool. Disease-specific comprehensive management programs and early input from palliative care teams are logical approaches. Despite recent publicity of end of life discussions, the message did not seem to change either patients’ or physicians’ behaviors. Identifying appropriate candidates and early discussion and implementation of end of life care will not only reduce readmission rates but will also improve symptom control and patient comfort toward end of life.

## Conclusions

Readmission rates will serve as quality measures influencing hospital and physician reimbursement. Patients, health care providers, and hospital systems share the responsibility for early revisits of the patient to hospital. Patients’ behavioral lifestyle choices, comorbid conditions, new unrelated diagnoses, and end-stage illnesses contribute to the majority of causes for readmission. Although most etiological factors for readmissions are universal to all hospitals, the relative contribution of each factor will be different in different settings. We therefore encourage medical centers to review individual institution data and develop specific plans to get the best return on their investments.

## Limitations

The study is retrospective in nature; however, with the comprehensive electronic medical record system and consistency in the methods of documentation at teaching services, we believe the data were adequate to produce valid results. By design, our study was limited to the patients on teaching services and may not be applicable to the general population.

Data extraction by individual reviewers serves as a potential for error. That said, efforts to minimize error and maintain consistency in data extraction included: (a) use of standardized categories of etiological factors, (b) restricting data analysis to teaching services, which in turn represents uniformity in documentation, and (c) adequately training individual reviewers and adopting similar methods for chart review. In addition, random reviews by supervising reviewers ensured accuracy of the extracted data.
